# Unravelling the causal link between gut microbiota and acne risk using a genetic approach

**DOI:** 10.1093/skinhd/vzaf077

**Published:** 2025-11-05

**Authors:** Fangyuan Cao, Natalia S Ogonowski, Santiago Díaz-Torres, Brittany L Mitchell, Puya Gharhakhani, Nicholas G Martin, Michael A Simpson, Jue-Sheng Ong, Miguel E Rentería

**Affiliations:** Brain & Mental Health Program, QIMR Berghofer, Brisbane, QLD, Australia; School of Biomedical Sciences, Faculty of Health, Medicine and Behavioural Sciences, The University of Queensland, Brisbane, QLD, Australia; Brain & Mental Health Program, QIMR Berghofer, Brisbane, QLD, Australia; School of Biomedical Sciences, Faculty of Health, Medicine and Behavioural Sciences, The University of Queensland, Brisbane, QLD, Australia; Brain & Mental Health Program, QIMR Berghofer, Brisbane, QLD, Australia; School of Biomedical Sciences, Faculty of Health, Medicine and Behavioural Sciences, The University of Queensland, Brisbane, QLD, Australia; Population Health Program, QIMR Berghofer, Brisbane, QLD, Australia; Brain & Mental Health Program, QIMR Berghofer, Brisbane, QLD, Australia; School of Biomedical Sciences, Faculty of Health, Medicine and Behavioural Sciences, The University of Queensland, Brisbane, QLD, Australia; School of Biomedical Sciences, Faculty of Health, Queensland University of Technology, Brisbane, QLD, Australia; School of Biomedical Sciences, Faculty of Health, Medicine and Behavioural Sciences, The University of Queensland, Brisbane, QLD, Australia; Population Health Program, QIMR Berghofer, Brisbane, QLD, Australia; School of Biomedical Sciences, Faculty of Health, Queensland University of Technology, Brisbane, QLD, Australia; Brain & Mental Health Program, QIMR Berghofer, Brisbane, QLD, Australia; School of Biomedical Sciences, Faculty of Health, Medicine and Behavioural Sciences, The University of Queensland, Brisbane, QLD, Australia; Department of Medical and Molecular Genetics, King’s College London, London, UK; School of Biomedical Sciences, Faculty of Health, Medicine and Behavioural Sciences, The University of Queensland, Brisbane, QLD, Australia; Population Health Program, QIMR Berghofer, Brisbane, QLD, Australia; Brain & Mental Health Program, QIMR Berghofer, Brisbane, QLD, Australia; School of Biomedical Sciences, Faculty of Health, Medicine and Behavioural Sciences, The University of Queensland, Brisbane, QLD, Australia; School of Biomedical Sciences, Faculty of Health, Queensland University of Technology, Brisbane, QLD, Australia

## Abstract

**Background:**

Acne is a common inflammatory dermatological condition that can have detrimental psychological consequences due to its visible lesions and scarring. Recent studies suggest a potential role of gut microbiota in acne development; however, the evidence remains inconclusive and might be subject to various confounders.

**Objectives:**

To investigate the causal relationship between gut microbiota and acne.

**Methods:**

This study investigated the causal relationship between gut microbiota and acne using a two-sample Mendelian randomization (MR) approach with large-scale genome-wide association study summary statistics. To ascertain the direction of causality and the independent effect of gut microbiota, reverse MR and multivariable MR accounting for dietary phenotypes were performed.

**Results:**

Higher abundances of the *Actinobacteria* phylum and class, *Bifidobacteriales* order, *Bifidobacteriaceae* family and *Bifidobacterium* ­genus were associated with a reduced risk of acne [odds ratios (ORs) ranging from 0.54 to 0.63]. In contrast, higher levels of the *Gastranaerophilales* order, *Streptococcaceae* family and *Streptococcus* genus were positively associated with an increased risk of acne (OR 1.12–1.36). Notably, associations for *Bifidobacterium* and its upstream taxa remained robust even after accounting for dietary factors.

**Conclusions:**

These findings provide further evidence of a causal relationship between gut microbial composition and acne, highlighting the role of gut microbiota in developing more targeted and possibly less harmful alternatives to current acne management strategies.

## Teaser

We used Mendelian randomization to show that certain gut bacteria, including *Bifidobacterium*, are linked to reduced acne risk, while others like *Streptococcus* are associated with increased risk. These findings strengthen evidence for a causal role of gut microbiota in acne development and suggest potential new avenues for targeted treatment.

What is already known about this topic?

- Acne remains one of the most common and psychologically impactful dermatological conditions, with increasing concern surrounding its management and treatment options.
- Previous observational studies and small-scale clinical trials have suggested a role for dysbiosis in acne pathogenesis, but causal evidence remains limited.

What does this study add?

- By triangulating human genetic data, our study supports a causal relationship between gut microbial composition and acne.
- These findings highlight gut microbiota as a clinically relevant and modifiable factor in acne and that microbiome-targeted interventions could become a promising therapeutic avenue in its prevention and treatment.

Acne is a highly prevalent, chronic inflammatory skin condition characterized by open and closed comedones, papules, pustules, nodules and cysts, and varying degrees of scars in sebum-rich body sites, including the face, neck, upper chest and back.^1^ It typically begins around puberty with a prolonged disease course and high rates of recurrence,^1^ and nearly everyone will experience some degree of acne by the age of 15–17 years.^2,3^ The prevalence remains high in early adulthood, affecting approximately 40–60% of people in their twenties^4,5^ and can persist into middle age.^4^ Although the physical symptoms of acne might be perceived as trivial and self-resolving, it can significantly affect multiple facets of life: the 2019 Global Burden of Disease Study estimated that acne accounted for ∼3.6 million disability-adjusted life years among individuals aged 10–24 years worldwide.^6^ Due to visible skin lesions, disfigurement and scarring, acne can be a source of emotional distress, fostering lower self-­esteem, isolation and negative body image,^7–9^ which significantly affect personal relationships, social engagement, and academic and professional performance,^10,11^ especially during the teenage years and early adulthood when appearance is pivotal in social interactions. Increased rates of anxiety, depression and other psychological sequelae have also been reported,^12,13^ with suicidal ideation being 2–3 times more common in people with acne than in those with minimal or no acne.^10^

The underlying pathogenesis and causes of acne are not fully understood. Genetic predisposition, sunlight exposure, dietary factors and lifestyle are implicated in the disease aetiology, although the evidence remains ambiguous. While various treatment options are available, including oral and topical antibiotics, retinoids and systemic therapies, these medications often come with side effects such as dryness, irritation, potential antibiotic resistance and teratogenicity, in the case of isotretinoin.^1,14^ Growing evidence suggests that intestinal microbiota may contribute to acne development and might offer potential therapeutic pathways.

The human gut microbiota performs essential metabolic and immune functions^15,16^ and may modulate the host’s skin homeostasis.^14,17^ Alterations in the gut microbial ecosystem or dysbiosis disrupt the intestinal barrier, allowing the migration of gut microbiota and their metabolites to the skin,^18^ and instigating systemic immune and inflammatory responses.^19^ These reactions can trigger or aggravate skin inflammation, leading to conditions like acne. Recent studies indicate a possible link between acne and distinct gut microbiota composition^20^ and lower microbial diversity.^21^ Moreover, certain gut bacteria, such as probiotics – live microorganisms that offer health benefits^22^ – may promote skin health and alleviate dermatological conditions through their antimicrobial, anti-inflammatory and immunomodulating properties.^14,18^ Nevertheless, existing evidence, primarily from observational studies, is susceptible to numerous confounders, which can bias results and hinder the ability to infer causation.

Mendelian randomization (MR) is a genetic-based instrumental-variable technique that circumvents the confounding issue, providing a more reliable estimate of causality between two phenotypes. It mimics randomized controlled trials and offers a complementary approach to strengthen causal inference by using genetic variants [e.g. single nucleotide polymorphisms (SNPs)] as instrumental variables (IVs), which are randomly allocated during meiosis and independent of confounders.^23^ In this study, we leveraged a two-sample MR (TSMR) approach to investigate the causal relationship between gut microbiota and acne. We used the genome-wide association study (GWAS) summary statistics data from the MiBioGen consortium and the largest acne GWAS meta-analysis to date. To further explore whether gut microbiota mediates the effect of diet on acne, we conducted multivariable MR (MVMR) using GWAS data on dietary phenotypes from UK Biobank.

## Materials and methods

**Figure 1:**
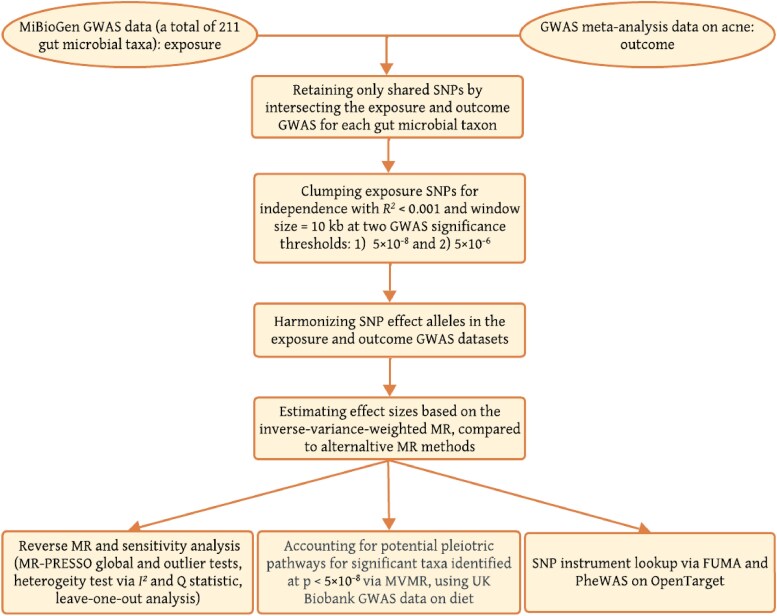
Workflow of instrumental selection and analysis process. This figure outlines the key steps involved in instrumental variable selection and statistical analysis. GWAS, genome-wide association study; MR, Mendelian randomization; MVMR, multivariable MR; PheWAS, phenome-wide association study; SNP, single nucleotide polymorphism.

In this study, we conducted a TSMR analysis to examine the causal relationship between gut microbiota and acne. We then performed reverse MR analyses to ascertain the direction of the causation. Finally, we assessed the direct influence of gut microbiota on acne, adjusting for dietary effects using MVMR. A range of sensitivity analyses were conducted to assess the validity of our results. An overview of the analysis workflow is depicted in Figure 1. This study leveraged publicly available summary-level data, and no ethical approval or informed consent was required. The reporting of this study adheres to the Strengthening the Reporting of Observational Study in Epidemiology MR (STROBE-MR) checklist.^24^

### Data sources

Data for gut microbiota were retrieved from the GWAS summary statistics provided by the MiBioGen international consortium.^25^ This is a large-scale, cross-ancestry GWAS meta-analysis of human genetics and gut microbiome that harmonized *16S* ribosomal RNA gene sequencing profiles and genotypes of 18 340 people across 24 cohorts. These included 16 European (*n* = 13 266), 1 Middle Eastern (*n* = 481), 1 East Asian (*n* = 811), 1 American Hispanic/Latin (*n* = 1097), 1 African American (*n* = 114) and 4 multiethnic cohorts (*n* = 2571).^25^ Microbiome quantitative trait loci mapping via Spearman correlation was performed to localize genetic loci associated with the abundance level (measured as the natural log-transformed read counts) of gut microbiota across 9 phyla, 16 classes, 20 orders, 35 families and 131 genera, which represent successive taxonomic ranks that group organisms from broad (phylum) to specific (genus) levels based on shared characteristics. Covariates, such as sex, age and top 10 genetic principal components, were consistently accounted for in all analyses.

For acne, data were sourced from the latest GWAS meta-analysis of 615 396 individuals (20 165 with acne and 595 231 without it) of European ancestry from 14 cohorts,^26^ namely the Avon Longitudinal Study of Parents and Children (ALSPAC), FinnGen, the Harvard Nurses’ Health Study (NHS; female only, initiated in 1976), NHS2 (female only, initiated in 1989), Health Professionals Follow-up Study (HPFS; male only, initiated in 1986), the Trøndelag Health Study (HUNT), the King’s College London (KCL) cohort, the Mass General Brigham Biobank, both the Teenage and Adult cohort from the Netherlands Twin Register, the Australian Genetics of Depression Study (AGDS), the Brisbane Longitudinal Twin Study (BLTS) and the Prospective Imaging of Aging Study (PISA) at QIMR Berghofer Medical Research Institute, and UK Biobank. Acne in these cohorts was ascertained by clinical assessment, health record or self-report. The final meta-analysis, utilizing an inverse-variance weighted (IVW) method, incorporated a total of 7 072 770 genetic variants that were available in at least 9 of the 14 cohorts.^26^ Detailed definitions of acne, recruitment strategy, genotyping and GWAS analysis for each cohort were described in the original publication.^26^

Data regarding dietary phenotypes were obtained from GWAS summary statistics of 445 779 participants from UK Biobank,^27^ a large population-based UK cohort aged 40–69 years.^28^ Dietary information was collected via a touchscreen questionnaire that assessed the weekly consumption frequency of various foods and beverages over the past year. In the GWAS analysis, food groups were identified via cluster analysis of different food items. These groups were represented by the first principal component, derived by a linear combination of the food traits within each cluster.^27^

### Genetic instruments

The GWAS summary statistics for each gut microbial taxon were merged with the full acne GWAS dataset, retaining only SNPs that were present in both datasets. From these shared SNPs, those associated with microbial abundance at a genome-wide significance threshold of *P* < 5 × 10^−8^ were selected. These SNPs were then clumped for independence using *PLINK* v1.90b6.8, with a linkage disequilibrium threshold of *R*^2^ < 0.001 and a window size of 10 000 based on the UK Biobank reference panel. Due to the limited number of variants reaching the conventional genome-wide significance level (*P* < 5 × 10^−8^) for many bacterial taxa, we additionally applied the same clumping procedure using a less stringent threshold of *P* < 5 × 10^−8^ as a secondary ­analysis. This approach increased the number of taxa with available genetic instruments, thereby enhancing our ability to explore potential associations.

The SNP instruments for gut microbiota and acne were harmonized to align effect alleles. Variants on the opposite strand or palindromic [inferred from the effect allele frequency (EAF)] were corrected by multiplying the effect size by −1. Multiallelic variants or those whose strand could not be confidently inferred (e.g. EAF close to 0.5) were excluded from the analysis.

The statistical power of MR analyses was assessed using the mRnd power calculator (https://cnsgenomics.com/shiny/mRnd/), based on the acne GWAS sample size and the phenotypic variance explained by the gut microbiota instruments. Our study has adequate power (80%) to detect an odds ratio (OR) as small as 1.15 per SD increase in microbial abundance.

### Statistical analysis

To assess the causal association between each individual microbial taxon and acne, we performed MR analysis using (i) the Wald ratio estimate when only a single SNP was available as instrument, and (ii) the IVW model when multiple SNPs were available. Reverse MR was performed for significant taxa, following the same procedure and instrument selection criteria described above, to explore the possibility of gut microbiota-acne association driven by reverse causality. The Benjamin–Hochberg false discovery rate (FDR) correction was applied to account for multiple tests, with an FDR-adjusted *P*-value of 0.05 defined as the level of statistical significance.

We adopted a series of sensitivity analyses to evaluate the validity of our MR estimates in the presence of potential violations of key assumptions:^23^ (i) the IVs are associated with the exposure; (ii) they are not associated with confounding variables; and (iii) they are associated with the outcome only through the exposure.

The strength of selected IVs for each bacterial taxon under both GWAS cutoffs (*P* < 5 × 10^−8^ and *P* < 5 × 10^−6^) was evaluated using the *F* statistic,^29^ which measures the proportion of variance of the trait explained by each IV. An *F* value greater than the empirical level of 10 indicates a strong instrument.

The second and third assumptions were checked via alternative MR approaches that rely on different assumptions. These methods included the weighted median estimator,^30^ which provides robust estimates and allows for the violation of the second and third MR assumptions when >50% of the weight is contributed by valid instruments; the weighted mode estimator,^31^ which robustly estimates the causal effect when the mode of or most common causal effect estimates come from valid instruments, even when the majority of SNPs used as instruments are invalid; and MR Egger,^32^ which allows for a nonzero intercept to test the degree of horizontal pleiotropy (MR-Egger intercept test *P* < 0.05). Consistent effect estimates across multiple methods provide strong evidence for the causal effect that is less likely to be biased.

MR-PRESSO analysis,^33^ which detects (via the MR-PRESSO global test at *P* < 0.05) and corrects (via the MR-PRESSO outlier test at *P* < 0.05) for horizontal pleiotropy by removing outliers, was also performed. A comparison of the causal estimates before and after the correction of outliers was then made using the MR-PRESSO distortion test with *P* < 0.05, indicating significant distortion. Heterogeneity across the instrument SNPs was assessed using the *I*^2^ index, Cochran’s Q statistic and leave-one-SNP-out analyses. To further evaluate potential pleiotropy of the selected microbial IVs across taxa, we assessed the number of IVs associated with more than one taxon.

Next, MVMR analyses were performed on significant gut microbes identified at the primary GWAS threshold of *P* < 5 × 10^−8^ to evaluate their potential mediating effects through diet. The analysis considered the influence of overall diet patterns (oily and nonoily fish, fresh and dried fruits, salad and cooked vegetables) and coffee consumption (ground, instant and decaffeinated coffee). The strength of IVs was evaluated using conditional ­*F* ­statistics.^34^

Finally, we conducted functional annotation of SNP instruments for significant taxa using FUMA^35^ and phenome-wide association studies (PheWAS) for the top SNPs identified in FUMA via queries on the OpenTarget^36^ platform, applying a Bonferroni-corrected significance cutoff (*P* < 0.05/number of phenotypes assessed).

All statistical analyses were performed using the *TwoSampleMR v0.6.6, MRPRESSO and Mendelian Randomization* packages in R software (v4.3.1).

## Results

### Univariable Mendelian randomization analyses using genome-wide association study thresholds of 5 × 10^−8^

**Figure 2:**
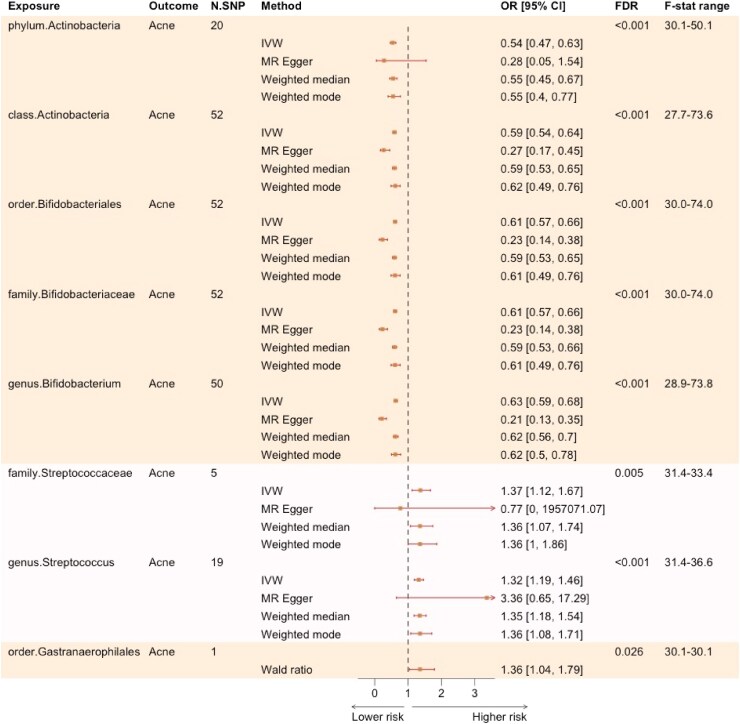
Effect estimates of significant gut microbiota in univariable Mendelian randomization (MR) at a genome-wide association study (GWAS) threshold of 5 × 10^−8^. This figure presents the significant associations between gut microbiota and acne at the primary significance threshold of 5 × 10^−8^, estimated using the inverse-variance weighted (IVW) method, in comparison with other MR methods. Microbial taxa are grouped by phylogenetic tree, with the same shade representing taxa within the same group. CI, confidence interval; FDR, false-discovery rate; N.SNP, number of single nucleotide polymorphism instruments; OR, odds ratio.

At the primary significance threshold of *P* < 5 × 10^−8^, 21 gut bacterial taxa had available SNPs, including 1 phylum, 1 class, 2 orders, 4 families and 13 genera (Table S1, see Supporting Information). Based on our IV selection criteria, the number of SNP instruments used in the MR analyses ranged from 1 to 52, with the *F* statistic for the corresponding SNPs well above 27 (Figure 2).

Five taxa with a positive effect on acne and three with a negative effect reached significance after FDR correction (Figure 2). The microbial taxa with abundance levels negatively associated with the risk of acne were the Actinobacteria phylum [OR_IVW_ 0.54, 95% confidence interval (CI) 0.47–0.63], the Actinobacteria class (OR_IVW_ 0.59, 95% CI 0.54–0.64), the Bifidobacteriales order (OR_IVW_ 0.61, 95% CI 0.57–0.66), the *Bifidobacteriaceae* family (OR_IVW_ 0.61, 95% CI 0.57–0.66) and the *Bifidobacterium* genus (OR_IVW_ 0.63, 95% CI 0.59–0.68; FDR < 0.001 for all). Gut microbes associated with increased acne risk included the Gastranaerophilales order (OR_IVW_ 1.36, 95% CI 1.04–1.79; FDR = 0.026), the *Streptococcaceae* family (OR_IVW_ 1.37, 95% CI 1.12–1.67; FDR = 0.005) and the *Streptococcus* genus (OR_IVW_ 1.32, 95% CI 1.19–1.46; FDR < 0.001).

The effect estimates were largely consistent across different MR methods. For the Actinobacteria phylum, *Streptococcaceae* family and *Streptococcus* genus, MR-Egger yielded less precise estimates, nonsignificant estimates; however, the direction of effect remained consistent. No significant genetic heterogeneity was observed for the IVW estimates (Table S2, see Supporting Information). The MR-Egger intercept test was significant (*P* < 0.05) for the Actinobacteria class, the Bifidobacteriales order, the *Bifidobacteriaceae* family and the *Bifidobacterium* genus. However, this is likely attributable to the large sample size in our analysis, and the intercept value was very small, with the ORs ranging from 1.05 to 1.1. In line with this assumption, the MR-PRESSO global test indicated no potential ­pleiotropy or outliers (Table S3, see Supporting Information). SNP IVs were specific to each taxonomic group, with no overlap observed across unrelated taxa (Table S4, see Supporting Information). Leave-one-SNP-out analyses showed no evidence that the association is driven by any SNP outliers. Lastly, no reverse associations (i.e. presence of acne resulting in changes in gut microbiota) were detected in the reverse MR.

### Univariable Mendelian randomization analyses using genome-wide association study thresholds of 5 × 10^−6^

**Figure 3:**
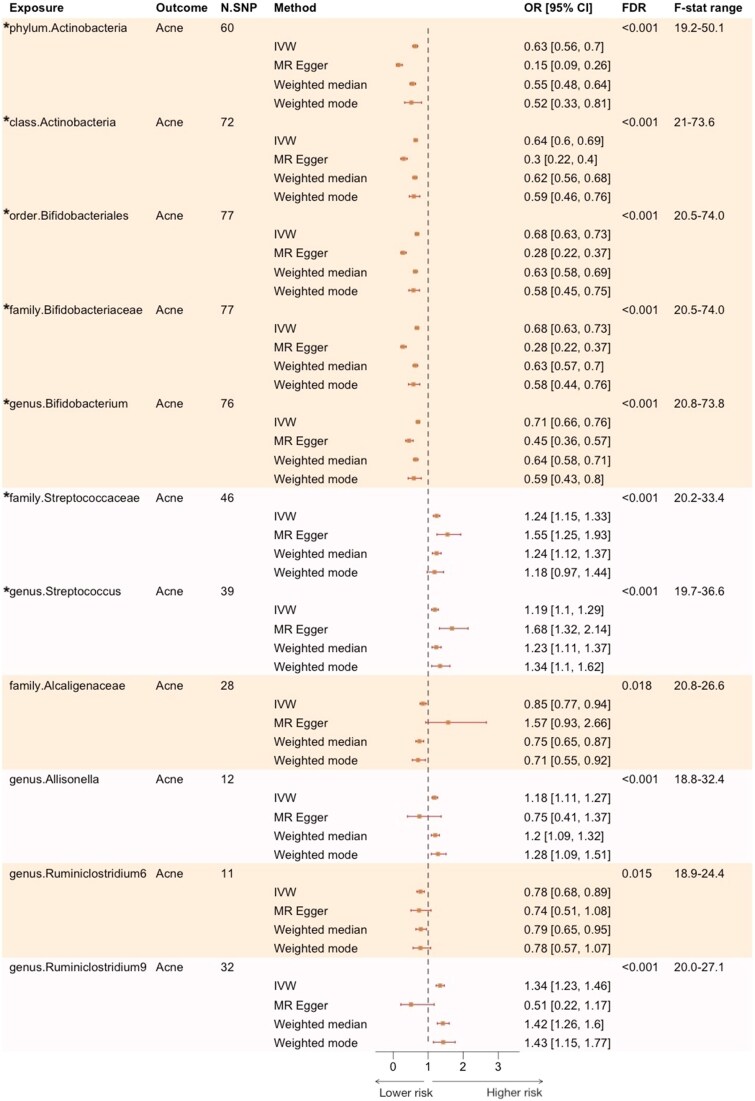
Effect estimates of significant gut microbiota in univariable Mendelian randomization at a genome-wide association study threshold of 5 × 10^−6^. This figure presents the significant associations between gut microbiota and acne at the secondary significance threshold of 5 × 10^−6^, estimated using the inverse-variance weighted (IVW) method, in comparison with other MR methods. Microbial taxa are grouped by phylogenetic tree, with the same shade representing taxa within the same group. An asterisk (*) denotes taxa that reached statistical significance at the primary significance threshold of 5 × 10^−8^. CI, confidence interval; FDR, false discovery rate; N.SNP, number of single nucleotide polymorphism instruments; OR, odds ratio.

At the secondary significance threshold of *P* < 5 × 10^−6^, more microbial taxa were available for MR analyses, including 9 phyla, 16 classes, 20 orders, 32 families and 120 genera (Table S5, see Supporting Information). Using the same IV selection criteria, the number of SNP instruments utilized ranged from 11 to 77, with the *F* statistic above 18 for all SNPs (Figure 3).

All significant taxa identified in the primary analysis were also observed in the secondary analysis, except for the Gastranaerophilales order. Additionally, new associations between gut microbiota and acne were identified, namely the *Alcaligenaceae* family (OR_IVW_ 0.85, 95% CI 0.77–0.94; FDR = 0.018), the genus *Ruminiclostridium 6* (OR_IVW_ 0.78, 95% CI 0.68–0.89; FDR = 0.015), the genus *Ruminiclostridium 9* (OR_IVW_ 1.34, 95% CI 1.23–1.46; FDR < 0.001) and *Allisonella* (OR_IVW_ 1.18, 95% CI 1.11–1.27; FDR < 0.001) (Figure 3).

The IVW estimates were consistent with alternative MR methods for most taxa, except for the family *Alcaligenaceae* and the genus *Ruminiclostridium 9*, where the MR-Egger effect estimates did not reach statistical significance. Cochran’s Q statistic revealed potential genetic heterogeneity for the phylum Actinobacteria and the genus *Bifidobacterium* (Table S6, see Supporting Information). Similarly, while the MR-Egger intercept test was significant for most significant taxa, except for the genus *Allisonella* and the genus *Ruminiclostridium 6*, the intercept effect sizes were very small (ORs ranged from 0.97 to 1.09). The MR-PRESSO global test (Table S7, see Supporting Information) and leave-one-SNP-out analyses showed no evidence of pleiotropy or outliers, and no overlapping SNP IVs were observed across different taxonomic groups (Table S8, see Supporting Information).

### Multivariable Mendelian randomization analyses

**Figure 4:**
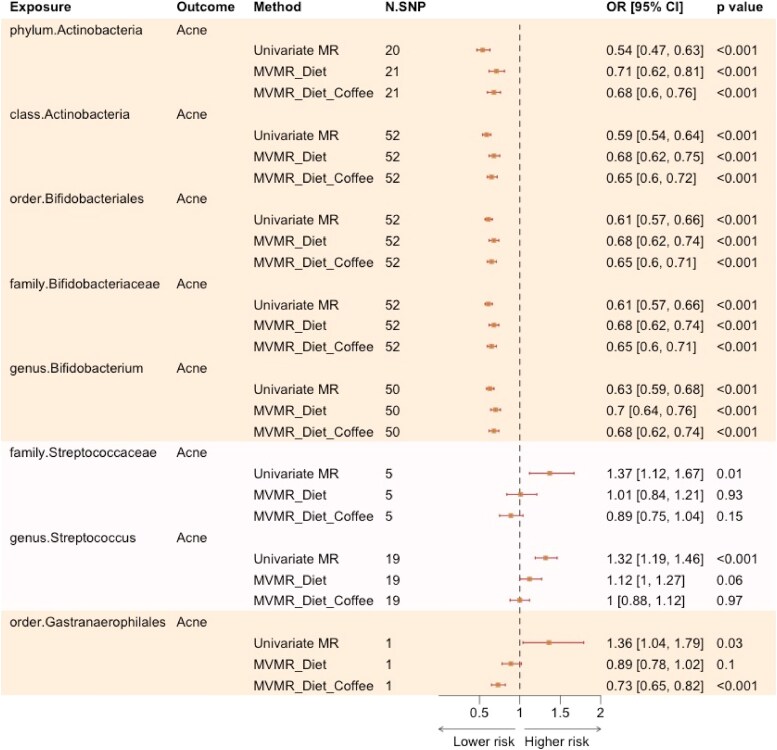
Effect estimates of gut microbiota in univariable vs. multivariable Mendelian randomization (MR) with dietary factors. This figure compares univariable MR effect estimates at the primary significance threshold of 5 × 10^−8^ with multivariable MR (MVMR) estimates, adjusting for overall diet pattern and overall diet pattern plus coffee consumption. Microbial taxa are grouped by phylogenetic tree, with the same shade representing taxa within the same group. CI, confidence interval; N.SNP, number of single nucleotide polymorphism instruments; OR, odds ratio.

MVMR analyses adjusting for dietary factors (e.g. overall diet patterns and/or coffee consumption) revealed robust causal (marginal) associations with acne risk for the Actinobacteria phylum and class, the Bifidobacteriales order, the *Bifidobacteriaceae* family and the *Bifidobacterium* genus (Figure 4). The marginal effect size estimates in MVMR were consistent with the univariable MR estimates, with only minor attenuations towards the null effect (an OR of 1). In comparison, the associations were no longer significant for the *Streptococcaceae* family and the *Streptococcus* genus [OR_IVW_ 1.01 (95% CI 0.84–1.21) and OR_IVW_ 1.12 (95% CI 1–1.27), respectively, after the inclusion of diet; OR_IVW_ 0.89 (95% CI 0.75–1.04) and OR_IVW_ 1 (95% CI 0.88– 1.12), respectively, after controlling for both diet and coffee intake]. For the *Gastranaerophilales* order, the association attenuated totally to the null after controlling for diet (OR_IVW_ 0.89, 95% CI 0.78–1.02) and were reversed in direction after controlling for both diet and coffee (OR_IVW_ 0.73, 95% CI 0.65–0.82).

Given the relatively small number of SNP instruments available for gut microbiota compared with diet variables, the conditional *F* statistic was <10 for all gut microbes (range 1–8) in the MVMR analysis that included diet patterns. When diet patterns and coffee intake were considered, the conditional *F* statistic fell below 10 for all exposures (range 1–7) due to the dilution effect of incorporating additional SNPs.

### Functional analyses and phenome-wide association studies

Finally, through FUMA and PheWAS, we probed evidence for horizontal pleiotropy by evaluating potential pleiotropic associations between our MR instruments and a wide range of human complex traits (Tables S9–S11, see Supporting Information). Results indicated that the top SNP instruments for the genus *Bifidobacterium* and its upstream taxa were mainly associated with blood cell levels (monocytes, neutrophils, eosinophils, and red and white blood cells) and lipid profiles (low-density and high-density lipoprotein cholesterol levels). Other traits included lactose intolerance, bread and coffee consumption, fat composition, testosterone levels and hair colour. The instrument for the *Streptococcus* genus and its family was enriched for traits related to mental health, such as depression and mood disorders. For the Gastranaerophilales order, the identified traits included feelings of worry or anxiety, red blood cell counts, cannabis use and wine intake.

## Discussion

In this study, we utilized large GWAS summary data to investigate the causal relationship between gut microbiota and acne via a TSMR approach. Our results indicated that higher abundance levels of the *Bifidobacterium* genus, along with its family *Bifidobacteriaceae*, order Bifidobacteriales and class, and phylum Actinobacteria, confer a protective effect against acne that is independent of dietary components in terms of fruit, vegetable, fish and coffee consumption. Conversely, increased abundance of the Gastranaerophilales order, the *Streptococcus* genus and its family *Streptococcaceae* is linked to a higher risk of acne. Using a relaxed significance threshold for SNP instrument inclusion, we identified several possible causal associations, where the *Alcaligenaceae* family and *Ruminiclostridium 6* genus may lower acne risk, whereas the *Allisonella* and *Ruminiclostridium 9* genera may elevate acne risk.

Our findings provide additional insights into the existing literature, which has presented mixed findings. One case–control study found that acne is associated with a depletion of *Bifidobacterium*, along with *Butyricicoccus*, *Coprobacillus*, *Lactobacillus* and *Allobaculum*, based on faecal samples from 31 individuals with moderate-to-severe acne and 31 control participants.^21^ In contrast, another study with a similar design observed diminished levels of the genus *Clostridia*, Clostridiales, *Lachnospiraceae* and *Ruminococcaceae* in people with acne compared with control participants (*n* = 43 in each group).^20^ This may be largely driven by the inability to accrue large sample sizes, as well as the potential bias and confounding inherent in observational studies. While there have been a few MR studies utilizing the MiBioGen (for gut microbiome) and FinnGen (for acne) GWAS summary, the findings remain challenging to interpret, with 3,^37^ 7^38^ and 16^39^ MR associations reported, respectively. These discrepancies likely stem from several methodological limitations: (i) all studies adopted a GWAS threshold of *P* < 1.0 × 10^−5^, raising concerns about the robustness of MR findings against weak instrument bias; and (ii) the FinnGen acne GWAS data, derived from only ∼3000 cases (compared with ours with >20 000 cases), which severely constrained the power of the study. In our analysis with a much larger case accrual for acne, only *Bifidobacterium* and *Streptococcus* at the genus level, along with their preceding taxa, and the Gastranaerophilales order, were uncovered as having causal impacts on acne at the standard GWAS significance threshold (*P* < 1.0 × 10^−8^). After accounting for potential sources of pleiotropy through diet intake, the effect of *Bifidobacterium* and its upstream taxa remained robust with only a slight attenuation towards the null, whereas other bacterial taxa appeared to exert their effects via interactions with diet.

*Bifidobacteria* are major probiotic bacteria that exert a range of beneficial effects on the host’s health, including the antimicrobial, anti-inflammatory and immunomodulatory actions, which may contribute to the prevention or improvement of acne. Although studies directly examining the effect of pro- and prebiotics on acne remain limited, it has previously been suggested that probiotic mixtures containing bifidobacteria may offer a synergistic anti-­inflammatory effect and mitigate adverse sequelae associated with systemic antibiotic therapy.^40^ Clinical trials and animal studies have shown that oral *bifidobacteria* not only can suppress ultraviolet-induced interleukin (IL)-1β in the skin and reduces transcutaneous water loss, skin dryness and epidermal thickening,^41^ but also improves skin barrier dysfunction and attenuate atopic dermatitis.^42^

Our PheWAS analyses revealed that the top SNP instruments for *Bifidobacterium* and its upstream taxa are associated with white blood cell counts, lipid profiles and testosterone levels. Elevated levels of white blood cells such as monocytes and neutrophils signal activation of the innate immune response and systemic inflammation, both of which are implicated in acne pathogenesis.^43^ Similarly, dyslipidaemia has been associated with acne both phenotypically, correlating with its severity,^44,45^ and genetically, with estimated genetic correlations of approximately −0.2 for triglyceride levels and −0.1 for cholesterol levels,^26^ potentially through its influence on androgen synthesis and sebum production.^44,45^ While it remains unclear whether these traits represent horizontal pleiotropy (confounding) or vertical pleiotropy (mediation) in the causal pathway linking microbiota to acne, converging evidence suggests that *Bifidobacterium* may contribute to acne through multiple interconnected biological mechanisms.

Mechanistically, bifidobacteria may attenuate the immune response involved in acne pathogenesis by altering cytokine production and immune cell trafficking. Their metabolites, such as short-chain fatty acids (SCFAs), play a significant role in immune modulation. For example, acetate has been shown to reduce neutrophil production and induces apoptosis,^46^ while butyrate enhances the expression of ­anti-inflammatory cytokines (e.g. IL-10).^47^

Bifidobacteria may also influence acne development by modulating insulin-like growth factor 1 (IGF-1), a key hormone linked to acne severity.^1^ Elevated IGF-1 promotes hyperkeratinization, proinflammatory cytokine secretion, sebocyte proliferation and lipogenesis,^48^ all of which contribute to the formation of comedones and a growth medium for acne-causing bacteria. Evidence suggests that bifidobacteria may regulate IGF-1 levels by improving insulin sensitivity,^49^ likely through preserving gut microbiota homeostasis, generating SCFAs and modulating metabolic signalling,^50–52^ including the mammalian target of the rapamycin pathway, which is upregulated in various inflammatory skin disorders.^53^

Furthermore, bifidobacteria can support balance gut microflora by inhibiting the adhesion or growth of pathogenic bacteria,^14,18^ thereby preventing systemic inflammation and immune dysregulation, and maintaining skin homeostasis.^54^ The robust association we observed between bifidobacteria and acne, independent of dietary factors, further supports a direct effect on acne or, at least, an effect that is not mediated by diet. Consistently, bifidobacteria supplementation has been found to improve glucose turnover rates and lower fasting insulin levels, even under high-fat dietary conditions.^54^

We also identified a modest association between the genus *Streptococcus* and its family *Streptococcaceae* and an increased risk of acne, with the MR estimated ORs ranging from 1.32 to 1.37 per SD increase in microbiota abundance level. However, this association diminished after adjusting for dietary patterns in the MVMR, implying that the effect of this microbial group might be mediated or moderated by diet. This hypothesis is supported by evidence from animal studies, which observed a marked reduction in *Streptococcus* abundance following 2-week high-fibre diet intervention.^55^

Functional annotation of the top SNP instruments for *Streptococcus* and its family revealed associations with neuropsychiatric traits, including depression and mood disorders. These conditions have previously been implicated in acne through clinical observations,^12,13^ showing modest genetic correlations with acne (e.g. ∼0.18 with schizophrenia and ∼0.1 with bipolar disorder and depression).^26^ These findings suggest a potentially shared genetic architecture or bidirectional interactions between gut microbial composition, acne and neuropsychiatric traits.

While streptococci showed consistent effect estimates across most alternative MR analyses, with no contradictory findings, the estimates from MR-Egger were ­nonsignificant. This may reflect inherent heterogeneity within this bacterial group. Streptococci are Gram-positive bacteria comprising diverse strains, ranging from harmless commensals to deleterious pathogens that interact with the host in myriad ways. Certain probiotic *Streptococcus* strains were found to benefit skin health and alleviate acne by increasing ceramide production, restoring the skin barrier and inhibiting the growth of *Cutibacterium acnes* via a bacteriocin-like inhibitory substance.^48^ Conversely, pathogenic strains such as group A *Streptococcus* are linked to invasive infections and inflammation of the skin and subcutaneous tissues.^56^ Thus, strain-level variability may obscure clearer associations, and further research is warranted to distinguish potentially beneficial from harmful subtypes.

Another risk-associated taxon identified was Gastranaerophilales, an order within the class Melainabacteria of the phylum Cyanobacteria. Gastranaerophilales was associated with an increased risk of acne in univariable MR, but this effect was reversed upon dietary adjustment, with the analysis being limited by the availability of a single SNP instrument. Our PheWAS analysis of this instrument suggested potential associations with emotional states (e.g. anxiety and worry), blood cell counts, and the use of cannabis and wine. Nevertheless, the phenotypic characterization of this bacterial group remains poorly defined with mixed findings. While Gastranaerophilales are believed to benefit their hosts via fermentation, serving as a source of vitamins B and K and aiding digestion,^57^ one study suggests that they may contribute to an increased risk of oropharyngeal cancer.^58^ Future research is needed to better understand the role of these microbes in human health and acne pathogenesis.

Beyond taxa identified at the primary GWAS significance threshold, our exploratory analyses using a more lenient threshold (*P* < 5 × 10^−6^) uncovered additional novel taxa potentially associated with acne, namely the *Alcaligenaceae* family belonging to the phylum Proteobacteria and the genera *Allisonella* and *Ruminiclostridium 6* and *9* of the phylum Firmicutes. Similarly, evidence on these gut microbes remains restricted and conflicting. *Ruminiclostridium* has been functionally implicated in various metabolic processes, including lipids, amino acids, carbohydrates, ­cofactors and vitamins metabolisms, as well as blood pressure regulation,^59^ which might have implications for skin health. However, certain *Ruminiclostridium* species have also been linked to an increased risk of ovarian cancer.^58^ In contrast, research on *Allisonella* and *Alcaligenaceae* is scarce, and their specific roles in human health remain elusive.

Our study extends the current understanding of gut microbiota and its relationship with acne in several ways. Leveraging very large-scale GWAS data for both gut microbiota abundance and acne susceptibility, we provided more robust evidence of their associations and revealed novel gut microbes that might potentially influence acne risks. Furthermore, to elucidate the direction, test the robustness of the MR estimates and explore the underlying causal mechanisms, we supplemented our main findings with reverse MR and MVMR analyses accounting for potential pleiotropic pathways, as well as evaluation of potential pleiotropy and biological plausibility through SNP instrument lookups via FUMA and PheWAS analyses.

However, our current analyses have several limitations. Although the international consortium GWAS represents the most comprehensive resource currently available for the gut microbiome, its sample size remains relatively small. Many taxa at the family and genus levels, such as *Lactobacillus* and *Butyricicoccus*, which have been highlighted in previous studies,^20,21^ cannot be assessed due to a lack of significant genetic variants, despite our attempt to circumvent this by relaxing the instrument selection criteria. The sequencing method in MiBioGen GWAS also limited the ability to assess gut microbiome at lower taxonomic levels (e.g. species and strains), which is crucial given the heterogeneity within even the same genus. For this reason, it is uncertain whether the observed effects of *Bifidobacterium* and its higher taxonomic groups, are driven by *Bifidobacterium* itself or by specific downstream species or strains. In addition, given that microbial abundance is typically measured in relative rather than absolute terms, the real-world interpretation of the effect sizes remains challenging. A fuller picture of this relationship may emerge as future microbiome GWAS continues to expand. The second limitation arises from the ancestral composition of the current datasets. The microbiome dataset includes multiple ancestries, although ∼72% were European, whereas the acne and diet datasets are restricted to European cohorts. Confounding due to multiancestry might not have been fully controlled for in the original GWAS analyses, and we were unable to conduct sensitivity analyses by separating European participants due to data limitations. Further investigations that better account for ancestral variation and potential confounders are needed.

Fermented foods (e.g. yoghurt), which might be implicated in the causal pathways for bacteria such as *Bifidobacterium*, were not queried in the UK Biobank food questionnaire and thus not accounted for in our MVMR analyses. We also acknowledge the broader limitations of self-reported dietary intake GWAS, which may be biased by reporting variability and cognitive and psychosocial factors. GWAS of objective dietary biomarkers (e.g. beta-carotene and caffeine metabolites), which remains limited in scale, could offer a valuable complementary perspective. Nonetheless, our results, along with previous findings,^21,40–42^ implicate that this bacterial group may exert direct effects on the skin and acne, independent of diet-related pathways. Lastly, the pathogenic process of acne involves a complex interplay of genetic predisposition and various environmental influences. Emerging research highlights the gut–brain–skin axis, linking gut microbiota, psychological distress and the skin microbiome in acne pathogenesis.^14,54^ One GWAS (*n* = 597) has attempted to investigate skin microbial traits; however, no loci reached study-wide significance.^60^ Due to the current lack of large-scale GWAS data, we are unable to evaluate these influences using genetic approaches. Future studies that integrate the skin microbiome profiling with genomic and phenotypic data will be essential to clarify these complex interactions.

This study, by leveraging human genetic data, provides additional evidence on the causal association between gut microbiota and acne. Our findings highlight that gut microbiota as a modifiable factor that may directly influence acne development, regardless of dietary impact. These insights not only extend our understanding of acne pathogenesis, but also inform future preventative and therapeutic strategies. However, further investigation with a larger sample size and more sophisticated sequencing techniques are warranted to allow for a comprehensive evaluation of the gut microbiome and a deeper understanding of specific microbes that are driving the effects.

## Supplementary Material

vzaf077_Supplementary_Data

## Data Availability

The GWAS summary statistics used in this study are publicly available and can be accessed through the following sources: gut microbiota GWAS data are available at www.mibiogen.org, diet phenotype GWAS data can be accessed in the GWAS catalogue with accession ID GCP000298 at http://ftp.ebi.ac.uk/pub/databases/gwas/summary_statistics/GCST90096001-GCST90097000/GCST90096892/ and acne GWAS data are available in the GWAS catalogue (https://www.ebi.ac.uk/gwas/) with accession number GCST90092000. Further details can be found in the references cited within this article.
